# Supplementary material for: Simple Adjustment of Intranucleotide Base-Phosphate Interaction in the OL3 AMBER Force Field Improves RNA Simulations
Source: J Chem Theory Comput. 2023 Nov 9;19(22):8423–33. doi: 10.1021/acs.jctc.3c00990 (PMC10687871; doi:10.1021/acs.jctc.3c00990)
Supplement: Supplementary file 1 — ct3c00990_si_001.pdf [file ct3c00990_si_001.pdf]

Supporting Information to Simple Adjustment of Intra-nucleotide Base-phosphate Interaction in OL3 AMBER Force Field Improves RNA Simulations

Vojtěch Mlýnský,[1] Petra Kührová,[1,2] Petr Stadlbauer,[1,2] Miroslav Krepl,[1,2] Michal Otyepka,[2,3] Pavel Banáš,[1,2,3] and Jiří Šponer [1]

[1] Institute of Biophysics of the Czech Academy of Sciences, Královopolská 135, 612 00 Brno, Czech Republic
[2] Czech Advanced Technology and Research Institute, CATRIN, Křížkovského 511/8, 779 00 Olomouc, Czech Republic
[3] IT4Innovations, VSB – Technical University of Ostrava, 17. listopadu 2172/15, 708 00 Ostrava-Poruba, Czech Republic

Table of Contents

SUPPORTING RESULTS 2

SUPPORTING TABLES 4

SUPPORTING FIGURES 11

REFERENCES 21

SUPPORTING RESULTS

Standard MD simulations of RNA and DNA motifs with the NBfix$_{0BPh}$ modification show no negative side-effects. We performed a set of unbiased MD simulations to investigate effects of the NBfix$_{0BPh}$ modification on a range of RNA systems and common DNA motifs (Table S2). We used the OL3$_{CP}$ and OL15 force fields (*ff*s) for RNA and DNA simulations, respectively (see Methods in the main text). The OL3$_{CP}$ RNA *ff* was corrected by the gHBfix19 potential[1] (all –NH…N– base-base interactions are strengthened and all –OH…OR– and –OH…OR– sugar – phosphate interactions are weakened; see Methods in main text for details). We also used the gHBfix19 for DNA duplex simulations, where only –NH…N– base-base interactions were strengthened in the same way as for RNA simulations (sugar – phosphate interactions were not affected due to missing –OH groups).

Initially, we simulated two RNA duplexes, i.e., decamer with the r(GCACCGUUGG) sequence (excised from the PDB ID 1QC0[2] structure) and r(UUAUAUAUAUAUAA)$_2$ tetradecamer (PDB ID 1RNA[3]). We performed two independent 5 μs-long standard MD simulations of each above-mentioned duplex with the NBfix$_{0BPh}$ modification and compared them with the control simulations, i.e., those with the same settings just without the NBfix$_{0BP}$ as control runs. Duplex simulations with the NBfix$_{0BPh}$ modification showed stable behavior, which resulted in similar averaged RMSD values (calculated from the starting structures) as those obtained from control simulations (1.1 ± 0.2 Å and 1.6 ± 0.4 Å for the decamer and tetradecamer, respectively). We observed decreased base-pair opening frequency of both GC pairs and AU base pairs at the end of helices in simulations with NBfix$_{0BPh}$ modification. Terminal base-pair breathing (fraying) was reduced by ~2% and ~11% for dodecamer (GC pairs) and tetradecamer (AU pairs), respectively (Table S5). We note that observed fraying results could be within the sampling limits of the simulations and that accurate (unambiguous) experimental data for the quantification of frayed structures are still not available for RNA sequences. However, the decreased probability of terminal base-pair fraying is expected to have positive effects on the behavior of RNA systems in MD simulations (see Refs. [1, 4] for further discussion about base-pair fraying). The analysis of helical base-pair parameters, i.e., those measuring orientation and displacement of Watson–Crick base pairs, showed that the NBfix$_{0BPh}$ did not affect simulated A-RNA duplexes. Averaged values of helical parameters from control and NBfix$_{0BPh}$-modified simulations are mostly within statistical errors (showing fluctuations, Table S5). The NBfix$_{0BPh}$ modification has visible effect on calculated inclination and base pair roll of both RNA duplexes, where we identified modest decreases of averaged values by ~1.6° and 0.6° for the inclination and roll parameters, respectively; Table S5). Note that the helical structure parameter inclination and base pair step parameter roll are mathematically interconnected.[5] In summary, the NBfix$_{0BPh}$ has minimal effect on the A-RNA structure and the terminal base-pairs of A-RNA duplexes appear to be slightly more stable.

Next, we performed two independent 5 μs-long simulations of Drew-Dickerson DNA dodecamer (the d(CGCGAATTCGCG) sequence, PDB ID 1BNA[6]) in both settings, i.e., with and without the NBfix$_{0BPh}$ modification. DNA duplex also revealed stable behavior upon the addition of NBfix$_{0BPh}$ with similar averaged RMSD as the control set of simulations (1.5 ± 0.3 Å). Helical base-pair parameters obtained from simulations with the NBfix$_{0BPh}$ modification and control simulations are entirely comparable (Table S6). The NBfix$_{0BPh}$ modification did result in noticeable change of the propeller parameter (averaged values are increased by ~0.9° in comparison with control simulations), but those differences are much smaller than statistical errors showing fluctuations (Table S6).

We then performed two independent 5 μs-long simulations of the kink-turn Kt-7 motif (see Methods in main text and Figure S7A) with the NBfix$_{0BPh}$ modification and compared them

with prolonged simulations from our previous work[1], i.e., simulations without the NBfix$_{0BPh}$ used as control sets (Table S2). We observed the H-bonds characterizing A-minor I interaction,[7] i.e., G6(2'-OH)…A16(N1), C11(2'-OH)…A16(O2'), and G6(N2H)…A16(N3), were lost in one simulation with the NBfix$_{0BPh}$ modification (~3 μs) and somewhat earlier during both control simulations (before ~1 us, Figures S8 and S9). In all three simulations, this loss was immediately followed by transition into A-minor 0 interaction, which is characterized by formation of G6(2'-OH)…A16(N3) and G6(O2')…A16(2'-OH) H-bonds (Figures S8 and S9). Transitions from A-minor I to A-minor 0 are well-known and occurring regularly in Kt-7 simulations, when this kink-turn is simulated out of its structural context.[8] We note the reversible attempts to transition from A-minor I into A-minor 0 were also observed during second simulation with the NBfix$_{0BPh}$ modification (Figure S9). In conclusion, the dynamics and stability of the Kt-7 motif was qualitatively same in NBfix$_{0BPh}$-modified and control simulations.

We then tested the NBfix$_{0BPh}$ modification on the Sarcin-Ricin loop RNA motif (see Methods in main text and Figure S7B). The overall SRL fold remained stable and we observed comparable dynamics between simulations with the NBfix$_{0BPh}$ modification and control runs (one prolonged simulation from our previous work[1] and one entirely new simulation, Table S2). We identified that the stability of one BPh contact, i.e., the G16(N1)…U8(O2P) H-bond, is weakened (presumably water-bridged) for most of the time in both NBfix$_{0BPh}$-modified and control simulation sets (Figures S10 and S11). Importantly, the structural stability of both the whole GpU platform, i.e., residues G7, U8 and A17, and the neighboring trans Hoogsteen/Sugar Edge A9G16 base pair were unaffected (Figures S10 and S11). Thus, SRL simulations with and without the NBfix$_{0BPh}$ modification provided basically identical outcome within the limits of sampling.

Finally, we investigated possible influence of the NBfix$_{0BPh}$ modification on structural behavior of the parallel-stranded (1KF1)[9] and (3+1) hybrid (2GKU)[10] DNA GQ's during 2 μs-long standard MD simulations. We observed very similar (basically identical within the limits of sampling) structural behavior of both tested GQ's in simulations with and without the NBfix$_{0BPh}$. Both cation binding sites inside the GQ channel were fully occupied in all the simulations. The distributions of G-quartet planarities, stacking distances and helical twists showed insignificant differences between the tested *ff* variants (Figures S12 and S13). Population of the state with bifurcated hydrogen bonds in G-quartets[1] differed by a few per cents between the *ff*s, but without a uniform trend, so we assume it was caused by the stochastic nature of MD simulations (Table S7). Altogether, the results indicate no effect of the NBfix$_{0BPh}$ modification on GQ simulation behavior.

In summary, no adverse structural side-effects of the NBfix$_{0BPh}$ modification were noticed in our testing set of standard simulations of common folded RNA and DNA motifs.

SUPPORTING TABLES

Table S1: Comparison of original and modified pairwise vdW parameters via the NBfix approach for atoms involved in 0BPh intra-nucleotide interactions, i.e., the NBfix 0BPh modification, for RNA $OL3_{CP}$ and DNA OL15 *ff*s.

Atom pairs	Original		NBfix 0BPh	
	R (Å) [a]	ε (kcal/mol) [b]	R (Å) [a]	ε (kcal/mol) [b]
H5…OR ($OL3_{CP}$), H5…OS (OL15)	3.1308	0.0505	2.8808	0.0505
H4…OR ($OL3_{CP}$), H4…OS (OL15)	3.1808	0.0505	2.9308	0.0505

[a] distance, where the Lennard-Jones potential for the interaction of atoms i and j, $R_{i,j}$, is exactly zero. Parameters for particular Lennard-Jones pairs derived as: $R_{i,j} = R_i + R_j$

[b] depth of the potential well for the interaction of atoms i and j, $\varepsilon_{i,j}$. Parameters for particular Lennard-Jones pairs derived as: $\varepsilon_{i,j} = \sqrt{(\epsilon_i + \varepsilon_j)}$

Table S2: Overview of all performed enhanced sampling and standard MD simulations. [a]

RNA Systems	*ff*	WAT	gHBfix	$NBfix_{0BPh}$	Method	Reps. [b]	Length [µs]
r(GACC) [c]	$OL3_{CP}$	OPC	gHBfix19	No	REST2	8	10
r(GACC) [d]	$OL3_{CP}$	OPC	gHBfix19, tHBfix20	No	REST2	8	10
r(GACC)	$OL3_{CP}$	OPC	gHBfix21, tHBfix20	No	REST2	8	10
r(GACC)	$OL3_{CP}$	OPC	gHBfix19	Yes	REST2	8	10
r(GACC)	$OL3_{CP}$	OPC	gHBfix19, tHBfix20	Yes	REST2	8	10
r(GACC)	$OL3_{CP}$	OPC	gHBfix21, tHBfix20	Yes	REST2	8	10
r(CAAU) [c]	$OL3_{CP}$	OPC	gHBfix19	No	REST2	8	10
r(CAAU) [d]	$OL3_{CP}$	OPC	gHBfix19, tHBfix20	No	REST2	8	10
r(CAAU)	$OL3_{CP}$	OPC	gHBfix21, tHBfix20	No	REST2	8	10
r(CAAU)	$OL3_{CP}$	OPC	gHBfix19	Yes	REST2	8	10
r(CAAU)	$OL3_{CP}$	OPC	gHBfix19, tHBfix20	Yes	REST2	8	10
r(CAAU)	$OL3_{CP}$	OPC	gHBfix21, tHBfix20	Yes	REST2	8	10
r(AAAA) [c]	$OL3_{CP}$	OPC	gHBfix19	No	REST2	8	10
r(AAAA) [d]	$OL3_{CP}$	OPC	gHBfix19, tHBfix20	No	REST2	8	10
r(AAAA)	$OL3_{CP}$	OPC	gHBfix21, tHBfix20	No	REST2	8	10
r(AAAA)	$OL3_{CP}$	OPC	gHBfix19	Yes	REST2	8	10
r(AAAA)	$OL3_{CP}$	OPC	gHBfix19, tHBfix20	Yes	REST2	8	10
r(AAAA)	$OL3_{CP}$	OPC	gHBfix21, tHBfix20	Yes	REST2	8	10
r(CCCC) [c]	$OL3_{CP}$	OPC	gHBfix19	No	REST2	8	10
r(CCCC) [d]	$OL3_{CP}$	OPC	gHBfix19, tHBfix20	No	REST2	8	10
r(CCCC)	$OL3_{CP}$	OPC	gHBfix21, tHBfix20	No	REST2	8	10
r(CCCC)	$OL3_{CP}$	OPC	gHBfix19	Yes	REST2	8	10
r(CCCC)	$OL3_{CP}$	OPC	gHBfix19, tHBfix20	Yes	REST2	8	10
r(CCCC)	$OL3_{CP}$	OPC	gHBfix21, tHBfix20	Yes	REST2	8	10
r(UUUU) [c]	$OL3_{CP}$	OPC	gHBfix19	No	REST2	8	10
r(UUUU) [d]	$OL3_{CP}$	OPC	gHBfix19, tHBfix20	No	REST2	8	10
r(UUUU)	$OL3_{CP}$	OPC	gHBfix21, tHBfix20	No	REST2	8	10
r(UUUU)	$OL3_{CP}$	OPC	gHBfix19	Yes	REST2	8	10
r(UUUU)	$OL3_{CP}$	OPC	gHBfix19, tHBfix20	Yes	REST2	8	10
r(UUUU)	$OL3_{CP}$	OPC	gHBfix21, tHBfix20	Yes	REST2	8	10
r(UCAAUC)	$OL3_{CP}$	OPC	gHBfix19	No	REST2	12	10
r(UCAAUC)	$OL3_{CP}$	OPC	gHBfix19, tHBfix20	No	REST2	12	10

r(UCAAUC)	OL3$_{CP}$	OPC	gHBfix21, tHBfix20	No	REST2	12	10
r(UCAAUC)	OL3$_{CP}$	OPC	gHBfix19	Yes	REST2	12	10
r(UCAAUC)	OL3$_{CP}$	OPC	gHBfix19, tHBfix20	Yes	REST2	12	10
r(UCAAUC)	OL3$_{CP}$	OPC	gHBfix21, tHBfix20	Yes	REST2	12	10
r(UCUCGU)	OL3$_{CP}$	OPC	gHBfix21, tHBfix20	No	REST2	12	10
r(UCUCGU)	OL3$_{CP}$	OPC	gHBfix19, tHBfix20	Yes	REST2	12	10
r(UCUCGU) [e]	OL3$_{CP}$	OPC	gHBfix19, tHBfix20	Yes	REST2	12	10
r(UCUCGU)	OL3$_{CP}$	OPC	gHBfix21, tHBfix20	Yes	REST2	12	10
gcGAGAgc [f]	OL3$_{CP}$	OPC	gHBfix19	No	ST-MetaD	12	5
gcGAGAgc	OL3$_{CP}$	OPC	gHBfix19	Yes	ST-MetaD	12	5
gcUUCGgc [f]	OL3$_{CP}$	OPC	gHBfix19	No	ST-MetaD	12	5
1QC0	OL3$_{CP}$	OPC	gHBfix19	No	sMD	1	5
1QC0	OL3$_{CP}$	OPC	gHBfix19	Yes	sMD	2	5
1RNA	OL3$_{CP}$	OPC	gHBfix19	No	sMD	1	5
1RNA	OL3$_{CP}$	OPC	gHBfix19	Yes	sMD	2	5
Kt-7 [g]	OL3$_{CP}$	OPC	gHBfix19	No	sMD	2	5
Kt-7	OL3$_{CP}$	OPC	gHBfix19	Yes	sMD	2	5
SRL [g]	OL3$_{CP}$	OPC	gHBfix19	No	sMD	2	5
SRL	OL3$_{CP}$	OPC	gHBfix19	Yes	sMD	2	5
r(CGCG)$_2$	*ff*99bsc0	TIP3P	-	No	sMD	1	1
r(CGCG)$_2$	*ff*99bsc0	TIP3P	-	Yes	sMD	1	1
r(CGCG)$_2$	*ff*99bsc0	OPC	-	No	sMD	1	1
r(CGCG)$_2$	*ff*99bsc0	OPC	-	Yes	sMD	1	1
r(CGCG)$_2$	OL3	TIP3P	-	No	sMD	1	1
r(CGCG)$_2$	OL3	TIP3P	-	Yes	sMD	1	1
r(CGCG)$_2$	OL3	OPC	-	No	sMD	1	1
r(CGCG)$_2$	OL3	OPC	-	Yes	sMD	1	1
r(CGCG)$_2$	*ff*99bsc0	TIP3P	gHBfix21	No	sMD	1	1
r(CGCG)$_2$	*ff*99bsc0	TIP3P	gHBfix21	Yes	sMD	1	1
r(CGCG)$_2$	*ff*99bsc0	OPC	gHBfix21	No	sMD	1	1
r(CGCG)$_2$	*ff*99bsc0	OPC	gHBfix21	Yes	sMD	1	1
r(CGCG)$_2$	OL3	TIP3P	gHBfix21	No	sMD	1	1
r(CGCG)$_2$	OL3	TIP3P	gHBfix21	Yes	sMD	1	1
r(CGCG)$_2$	OL3	OPC	gHBfix21	No	sMD	1	1
r(CGCG)$_2$	OL3	OPC	gHBfix21	Yes	sMD	1	1
1QC0	*ff*99bsc0	TIP3P	-	No	sMD	1	1
1QC0	*ff*99bsc0	TIP3P	-	Yes	sMD	1	1
1QC0	*ff*99bsc0	OPC	-	No	sMD	1	1
1QC0	*ff*99bsc0	OPC	-	Yes	sMD	1	1
1QC0	OL3	TIP3P	-	No	sMD	1	1
1QC0	OL3	TIP3P	-	Yes	sMD	1	1
1QC0	OL3	OPC	-	No	sMD	1	1
1QC0	OL3	OPC	-	Yes	sMD	1	1
r(CGCG)$_2$	*ff*99bsc0	TIP3P	gHBfix21	Yes	sMD	1	1
r(CGCG)$_2$	*ff*99bsc0	OPC	gHBfix21	No	sMD	1	1
r(CGCG)$_2$	*ff*99bsc0	OPC	gHBfix21	Yes	sMD	1	1
r(CGCG)$_2$	OL3	TIP3P	gHBfix21	No	sMD	1	1
r(CGCG)$_2$	OL3	TIP3P	gHBfix21	Yes	sMD	1	1
r(CGCG)$_2$	OL3	OPC	gHBfix21	No	sMD	1	1
r(CGCG)$_2$	OL3	OPC	gHBfix21	Yes	sMD	1	1
DNA Systems	*ff*	WAT	gHBfix	NBfix$_{0BPh}$	Method	Reps.[b]	Length [μs]
1BNA	OL15	SPC/E	gHBfix19 [h]	No	sMD	2	5
1BNA	OL15	SPC/E	gHBfix19 [h]	Yes	sMD	2	5
1KF1	OL15	SPC/E	-	No	sMD	1	2
1KF1	OL15	SPC/E	-	Yes	sMD	1	2
2GKU	OL15	SPC/E	-	No	sMD	1	2
2GKU	OL15	SPC/E	-	Yes	sMD	1	2

[a] See Methods in main text for details about system and *ff* labelling, description of HBfix potentials and setting of the NBfix$_{0BPh}$ modification.

[b] Number of replicas in enhanced sampling simulations (REST2 / ST-MetaD) or number of independent simulations for standard MD (sMD) simulations.
[c] Data taken from Ref. [1].
[d] Data taken from Ref. [11].
[e] REST2 simulation run with unbiased replica shifted from 298 K to 275 K (to mimic the experimental temperature).
[f] Data taken from Ref. [12].
[g] One (SRL motif) or two (Kt-7 motif) 1 μs-long MD simulations from Ref. [1] were prolonged to 5 μs. We performed one entirely new independent simulation of the SRL motif.
[h] The –NH…N– base-base interactions were stabilized by 1.0 kcal/mol (i.e., identically to gHBfix19 for RNA; destabilization of sugar-phosphate H-bonding is not applicable for DNA).

Table S3: Population of the C2'-endo pucker of r(UCUCGU) HN residues from simulations and experiment. [a]

r(UCUCGU)	Fraction of C2'-endo-like sugar pucker	
	REST2	Exp. [b]
U1	0.21	0.25
C2	0.13	0.25
U3	0.62	0.37
C4	0.59	0.28
G5	0.12	0.55
U6	0.50	0.43

[a] Data taken from REST2 simulation with $OL3_{CP}$, gHBfix19 and tHBfix20 potentials and with the $NBfix_{0BPh}$ modification (see Methods in main text and Table S2).
[b] Experimental data measured at 275 K.[13]

Table S4: Occurrence of spurious ladder-like structures in simulations of RNA duplexes. [a]

System	*ff* version	Correction	Water model	Fraying, top pair [%] [b]	Fraying, bottom pair [%] [b]	Ladder [ns]
CGCG	*ff*99bsc0	-	TIP3P	~24	~35	~7
	*ff*99bsc0	-	OPC	~70	~53	~12
	*ff*99bsc0	$NBfix_{0BPh}$	TIP3P	~95	~93	~13 – ~49 [c]
	*ff*99bsc0	$NBfix_{0BPh}$	OPC	~43	~75	~45 – ~85, ~335
	OL3	-	TIP3P	~57	~25	-
	OL3	-	OPC	~94	~26	~764
	OL3	$NBfix_{0BPh}$	TIP3P	~47	~10	-
	OL3	$NBfix_{0BPh}$	OPC	~27	~16	-
CGCG	*ff*99bsc0	gHBfix21	TIP3P	~0	~1	~504
	*ff*99bsc0	gHBfix21	OPC	~0	~0	-
	*ff*99bsc0	gHBfix21+$NBfix_{0BPh}$	TIP3P	~33	~7	-
	*ff*99bsc0	gHBfix21+$NBfix_{0BPh}$	OPC	~0	~0	-
	OL3	gHBfix21	TIP3P	~0	~0	-
	OL3	gHBfix21	OPC	~0	~3	-
	OL3	gHBfix21+$NBfix_{0BPh}$	TIP3P	~0	~0	-
	OL3	gHBfix21+$NBfix_{0BPh}$	OPC	~1	~0	-
1QC0	*ff*99bsc0	-	TIP3P	~0	~83	~150
	*ff*99bsc0	-	OPC	~3	~10	-
	*ff*99bsc0	$NBfix_{0BPh}$	TIP3P	~0	~2	-
	*ff*99bsc0	$NBfix_{0BPh}$	OPC	~1	~9	-
	OL3	-	TIP3P	~0	~0	-
	OL3	-	OPC	~65	~1	-

	OL3	$NBfix_{0BPh}$	TIP3P	~0	~0	-
	OL3	$NBfix_{0BPh}$	OPC	~0	~0	-
CGCG	*ff*99bsc0 [d]	gHBfix21	OPC	~0	~0	-
	*ff*99bsc0 [d]	gHBfix21+$NBfix_{0BPh}$	TIP3P	~1	~1	-
	*ff*99bsc0 [d]	gHBfix21+$NBfix_{0BPh}$	OPC	~0	~0	-
	OL3 [d]	gHBfix21	TIP3P	~9	~2	~98 [e]
	OL3 [d]	gHBfix21	OPC	~14	~0	~167 [e]
	OL3 [d]	gHBfix21+$NBfix_{0BPh}$	TIP3P	~7	~1	~237 [e]
	OL3 [d]	gHBfix21+$NBfix_{0BPh}$	OPC	~0	~1	~189 [e]

[a] All simulations were run for 1 μs (see Methods in main text and Table S2).
[b] Top and bottom pairs are denoted to residues C1G8 and G4C5 for $r(CGCG)_2$ duplex and G1C20 and G10C11 for 1QC0 duplex, respectively.
[c] We observed unfolding, i.e., strand separation, of the duplex structure at ~557 ns.
[d] Simulations were initiated form the ladder-like structure.
[e] Time denotes complete transition back to the canonical A-form duplex, i.e., reparation of the ladder-like structure.

Table S5: Averaged values of helical parameters (and standard deviations represented by error bars) and base pair fraying of 1QC0 and 1RNA duplexes. [a]

	Inclination [°]	Slide [°]	Twist [°]	Rise [Å]	Tip [°]	Tilt [°]	Shift [Å]	Buckle [°]	Opening [°]	Shear [Å]	Stagger [Å]	Stretch [Å]	Propeller [°]	Roll [°]	Minor groove [Å]	Major groove [Å]	Fraying (top, bottom) [b] [%]	
1QC0 (Exp.)	15.2	-1.7	30.8	2.7	2.1	-1.2	-0.1	1.2	1.4	0.1	0.1	-0.3	-12.5	8.1	13.1	18.1	-	-
gHBfix19	14.3 ± 0.7	-1.8 ± 0.1	31.3 ± 0.4	2.7 ± 0.0	-0.6 ± 0.3	0.3 ± 0.1	0.0 ± 0.0	1.5 ± 0.9	0.1 ± 0.2	0.0 ± 0.1	-0.1 ± 0.0	0.0 ± 0.0	-12.3 ± 0.7	7.7 ± 0.4	13.4 ± 0.0	19.2 ± 0.0	4.2	0.1
gHBfix19 + NBfix$_{0BPh}$	12.9 ± 0.5	-1.6 ± 0.1	31.2 ± 1.2	2.8 ± 0.1	-0.5 ± 0.1	0.3 ± 0.1	0.0 ± 0.0	1.3 ± 0.2	-0.2 ± 0.1	0.0 ± 0.0	-0.3 ± 0.0	0.0 ± 0.0	-13.7 ± 0.5	7.3 ± 0.3	13.4 ± 0.5	18.7 ± 0.7	0.1	0.0
	12.9 ± 0.5	-1.6 ± 0.6	31.3 ± 1.2	2.8 ± 0.1	-0.5 ± 0.1	0.3 ± 0.0	0.0 ± 0.0	1.3 ± 0.2	-0.3 ± 0.1	0.0 ± 0.0	-0.2 ± 0.0	0.0 ± 0.0	-13.8 ± 0.6	7.4 ± 0.3	13.4 ± 0.5	18.7 ± 0.7	0.1	0.1
1RNA(Exp.)	18.8	-1.3	30.6	2.6	-0.4	0.1	0.1	1.3	-0.4	-0.1	0.0	-0.2	-18.8	10.0	13.3	18.3	-	-
gHBfix19	18.1 ± 0.5	-1.5 ± 0.0	30.2 ± 0.1	2.5 ± 0.0	0.0 ± 0.1	0.0 ± 0.1	0.0 ± 0.0	0.0 ± 0.2	0.8 ± 0.2	0.0 ± 0.0	0.1 ± 0.0	0.0 ± 0.0	-17.5 ± 0.2	10.5 ± 0.3	13.3 ± 0.0	19.2 ± 0.0	22.4	7.1
gHBfix19 + NBfix$_{0BPh}$	16.4 ± 0.6	-1.3 ± 0.1	31.6 ± 1.2	2.6 ± 0.1	0.0 ± 0.1	0.0 ± 0.0	0.0 ± 0.0	0.0 ± 0.1	0.9 ± 0.1	0.0 ± 0.0	0.1 ± 0.0	0.0 ± 0.0	-17.3 ± 0.7	9.7 ± 0.4	13.3 ± 0.5	18.7 ± 0.7	2.5	3.1
	16.4 ± 0.6	-1.3 ± 0.1	31.5 ± 1.2	2.6 ± 0.1	0.0 ± 0.1	0.0 ± 0.0	0.0 ± 0.0	0.0 ± 0.2	0.9 ± 0.1	0.0 ± 0.0	0.1 ± 0.0	0.0 ± 0.0	-17.3 ± 0.7	9.8 ± 0.4	13.3 ± 0.5	18.7 ± 0.7	4.6	3.7

[a] All simulation were run in OL3$_{CP}$ *ff*, OPC water model and 0.15 M KCl (see Methods in main text and Table S2). The first two base pairs from each termini were excluded to prevent end effects and standard deviations were calculated using block average over 1000 snapshots (saved every 10 ps).

[b] Top and bottom pairs are denoted to residues G1C20 and G10C11 for 1QC0 duplex and U1A28 and A14U15 for 1RNA duplex, respectively.

Table S6: Averaged values of helical parameters (and standard deviations represented by error bars) and base pair fraying of 1BNA duplex. [a]

	Inclination [°]	Slide [°]	Twist [°]	Rise [Å]	Tip [°]	Tilt [°]	Shift [Å]	Buckle [°]	Opening [°]	Shear [Å]	Stagger [Å]	Stretch [Å]	Propeller [°]	Roll [°]	Minor groove [Å]	Major groove [Å]	Fraying (top, bottom) [b] [%]	
1BNA (Exp.)	0.4	0.1	35.6	3.3	0.0	-0.2	-0.1	-0.7	0.1	0.1	0.1	-0.2	-13.6	-0.3	12.3	18.9	-	-
gHBfix19	2.5 ± 5.1	0.0 ± 0.4	35.7 ± 2.9	3.3 ± 0.1	-0.1 ± 3.5	0.0 ± 2.1	0.0 ± 0.4	0.0 ± 4.6	-0.4 ± 0.7	0.0 ± 0.1	0.0 ± 0.1	-0.0 ± 0.0	-10.1 ± 7.3	1.3 ± 2.9	12.2 ± 0.2	19.2 ± 0.3	1.7	60.6
	2.6 ± 5.2	0.0 ± 0.4	35.6 ± 3.1	3.3 ± 0.1	0.0 ± 3.1	0.0 ± 1.9	0.0 ± 0.4	0.0 ± 4.6	-0.5 ± 0.7	0.0 ± 0.1	0.1 ± 0.1	0.0 ± 0.0	-10.8 ± 6.1	1.3 ± 2.9	12.2 ± 0.2	19.2 ± 0.4	0.1	0.8
gHBfix19 + NBfix$_{0BPh}$	1.8 ± 5.3	0.0 ± 0.4	35.8 ± 3.5	3.3 ± 0.1	0.1 ± 3.7	-0.1 ± 2.3	0.0 ± 0.4	-0.1 ± 4.8	-0.5 ± 0.8	0.0 ± 0.1	0.1 ± 0.1	0.0 ± 0.0	-9.5 ± 7.6	0.8 ± 2.9	12.2 ± 0.2	19.1 ± 0.4	69.2	1.3
	1.8 ± 5.3	0.0 ± 0.4	35.8 ± 3.5	3.3 ± 0.1	-0.1 ± 3.9	0.0 ± 2.3	0.0 ± 0.4	0.1 ± 4.8	-0.5 ± 0.8	0.0 ± 0.1	0.1 ± 0.1	0.0 ± 0.0	-9.6 ± 7.4	0.8 ± 2.9	12.2 ± 0.2	19.1 ± 0.4	0.1	62.0

[a] All simulation were run in OL15 *ff*, SPC/E water model and 0.15 M KCl (see Methods in main text and Table S2). The first two base pairs from each termini were excluded to prevent end effects and standard deviations were calculated using block average over 1000 snapshots (saved every 10 ps).
[b] Top and bottom pairs are denoted to residues C1G24 and G12C13, respectively.

Table S7: Population fraction of normal, bifurcated and other states of the middle quartet observed in the simulations of 1KF1 and 2GKU GQs. [a]

Simulation	Normal state	Bifurcated state	Other state
1KF1	0	0.948	0.052
1KF1, NBfix$_{0BPh}$	0	0.918	0.082
2GKU	0	0.948	0.052
2GKU, NBfix$_{0BPh}$	0	0.961	0.039

[a] All simulation were run in OL15 *ff*, SPC/E water model and 0.15 M KCl (see Methods in main text and Table S2).

SUPPORTING FIGURES

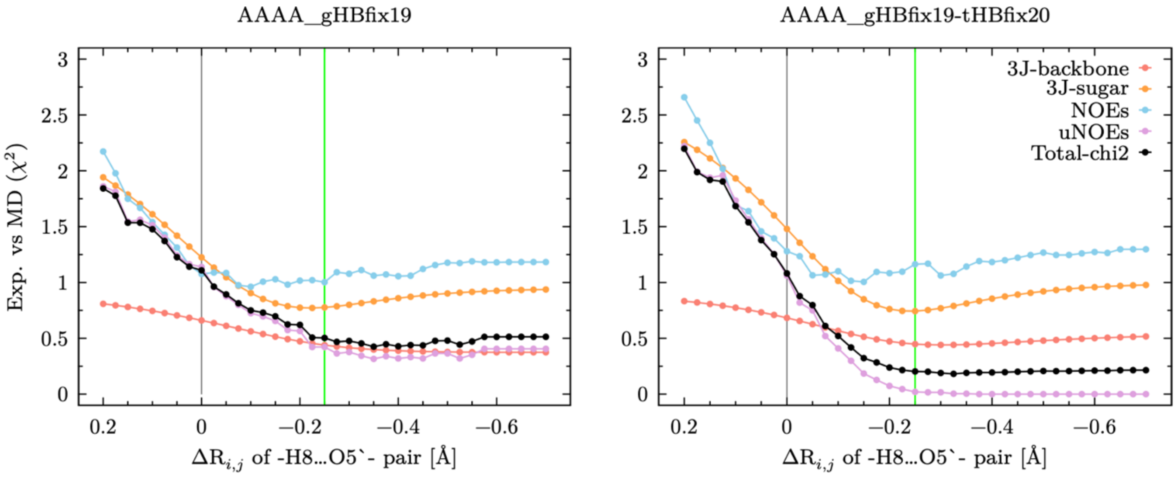

Figures S1: Effects of different settings of the $NBfix_{0BPh}$ modification on agreement between simulations and NMR data as probed by reweighting of two r(AAAA) TN trajectories from REST2 simulations (only trajectories from reference, i.e., unbiased, replicas were considered for the analysis). Evolution of NMR observables, i.e., ^{3}J scalar couplings, NOEs, uNOEs, and total χ^2 values (combination of all observables; see Methods in main text) from $OL3_{CP}$ REST2 simulations with gHBfix19 (panel A) and gHBfix19+tHBfix20 (panel B). Plots show dependence of NMR observables on changes of the Lennard-Jones $R_{i,j}$ parameter for purines, i.e., the distance between –H8…O5’– pairs, from the original (standard AMBER *ff*) values. Grey and green vertical lines mark positions of original and modified (i.e., chosen for further testing) parameter values, respectively.

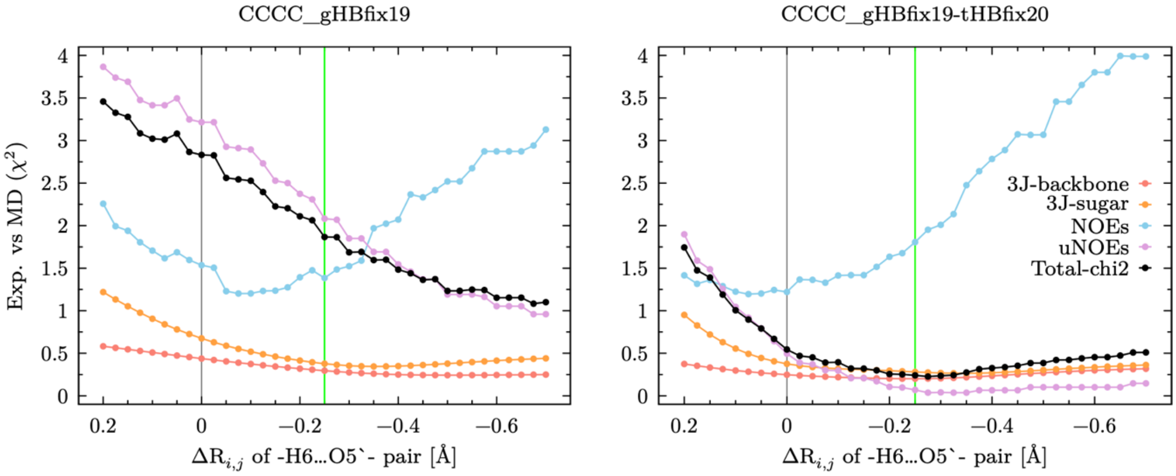

Figure S2: Effects of different settings of the $NBfix_{0BPh}$ modification on agreement between simulations and NMR data as probed by reweighting of two r(CCCC) TN trajectories from REST2 simulations. Plots show dependence of NMR observables on changes of the Lennard-Jones $R_{i,j}$ parameter for pyrimidines, i.e., the distance between –H6…O5’– pairs, from the original (standard AMBER *ff*) values. See legend of Figure S1 for more details.

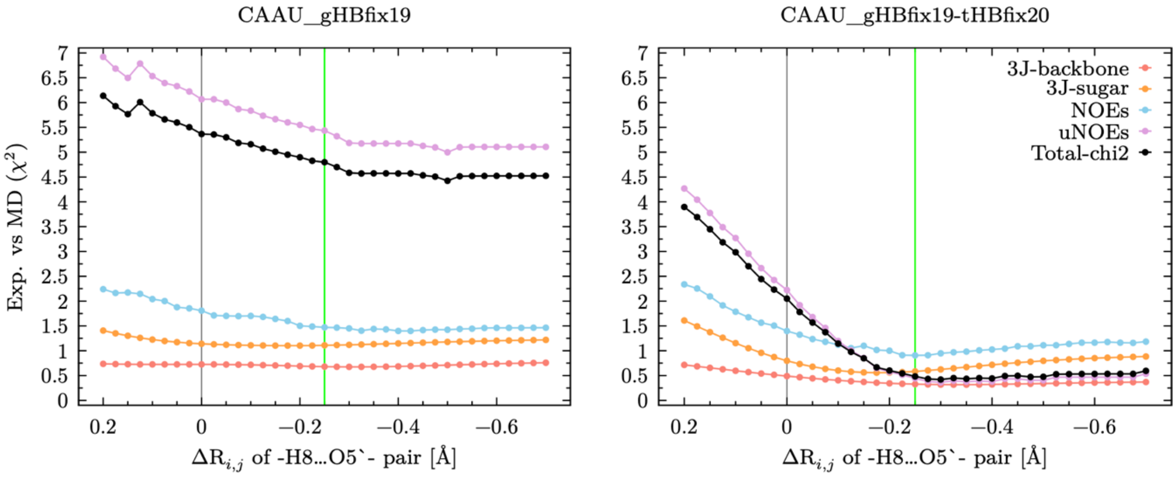

Figure S3: Effects of different settings of the NBfix$_{0BPh}$ modification on agreement between simulations and NMR data as probed by reweighting of two r(CAAU) TN trajectories from REST2 simulations. Plots show dependence of NMR observables on changes of the Lennard-Jones $R_{i,j}$ parameter for purines, i.e., the distance between –H8…O5'– pairs, from the original (standard AMBER *ff*) values. See legend of Figure S1 for more details.

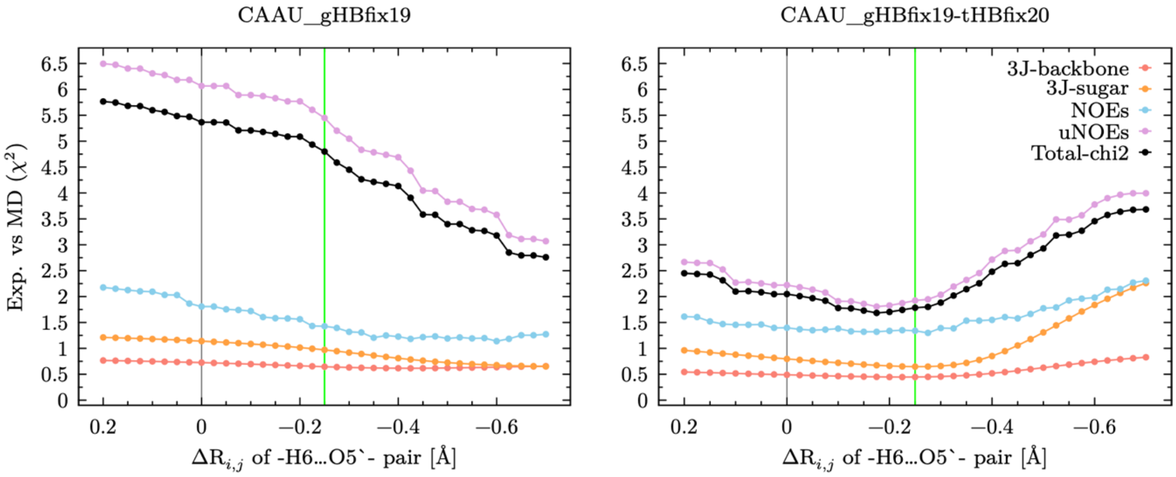

Figure S4: Effects of different settings of the NBfix$_{0BPh}$ modification on agreement between simulations and NMR data as probed by reweighting of two r(CAAU) TN trajectories from REST2 simulations. Plots show dependence of NMR observables on changes of the Lennard-Jones $R_{i,j}$ parameter for pyrimidines, i.e., the distance between –H6…O5'– pairs, from the original (standard AMBER *ff*) values. See legend of Figure S1 for more details.

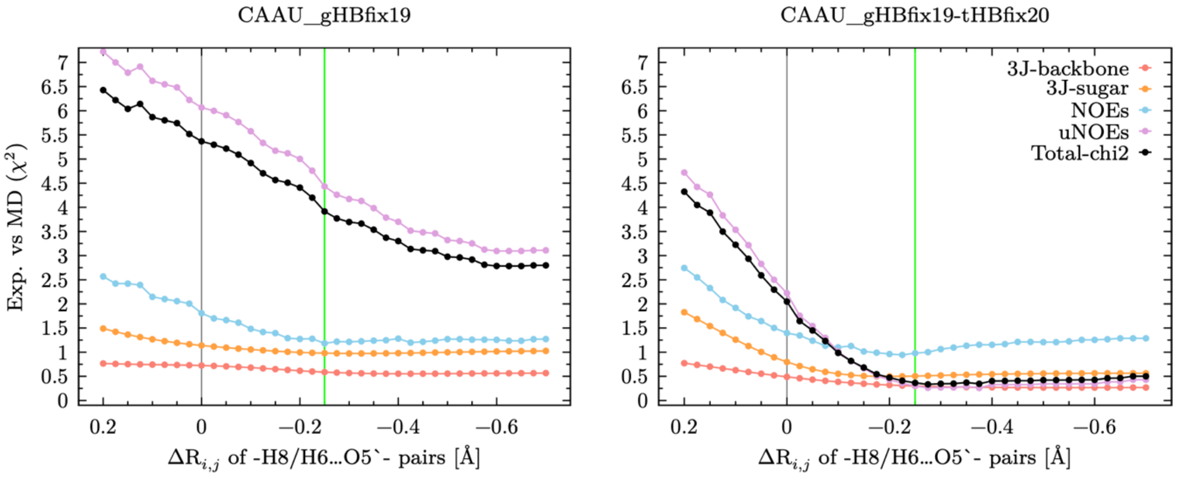

Figure S5: Effects of different settings of the NBfix$_{0BPh}$ modification on agreement between simulations and NMR data as probed by reweighting of two r(CAAU) TN trajectories from REST2 simulations. Plots show dependence of NMR observables on changes of the Lennard-Jones $R_{i,j}$ parameter for both purines and pyrimidines, i.e., distances between –H8…O5’– pairs and –H6…O5’– pairs, respectively, from the original (standard AMBER *ff*) values. See legend of Figure S1 for more details.

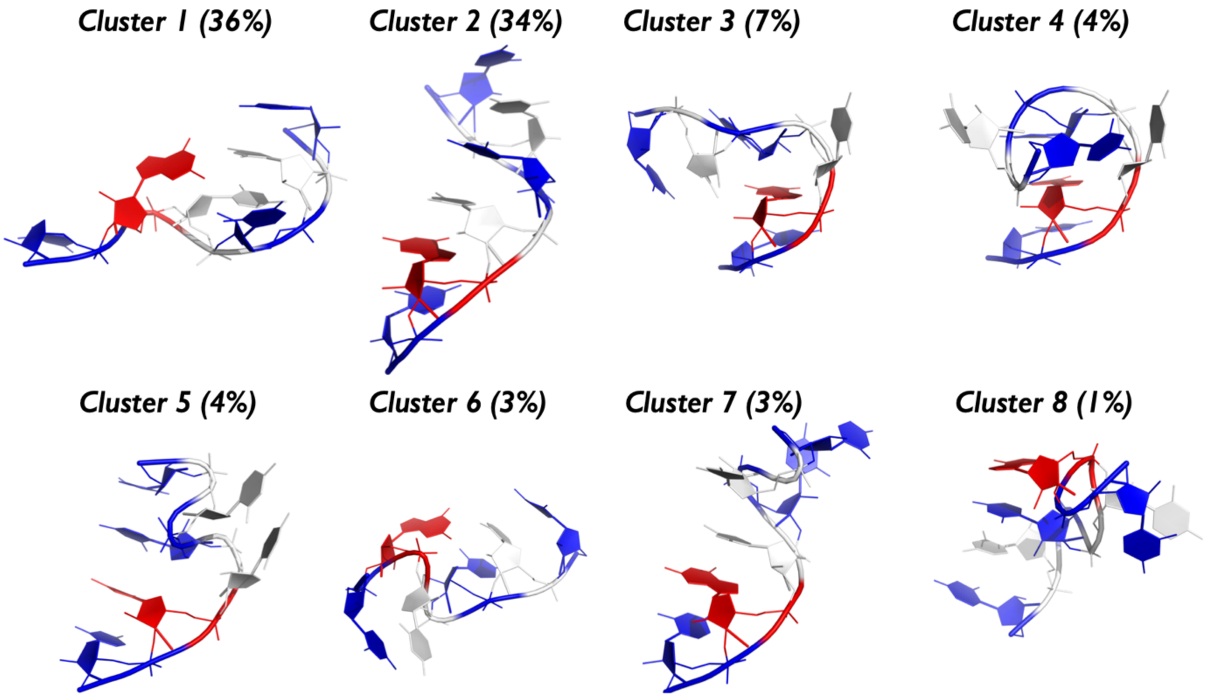

Figure S6: Tertiary structures of all populated clusters from r(UCUCGU) REST2 simulation with the OL3$_{CP}$ RNA *ff*, gHBfix19 and tHBfix20 potentials and the NBfix$_{0BPh}$ modification. C, G and U nucleotides are colored in white, red, and blue, respectively. H-atoms, ions and water molecules are not shown for clarity. Most populated conformers are loop-like state with G5C2 base pair and canonical A-form state. Population of unassigned structures is ~8%.

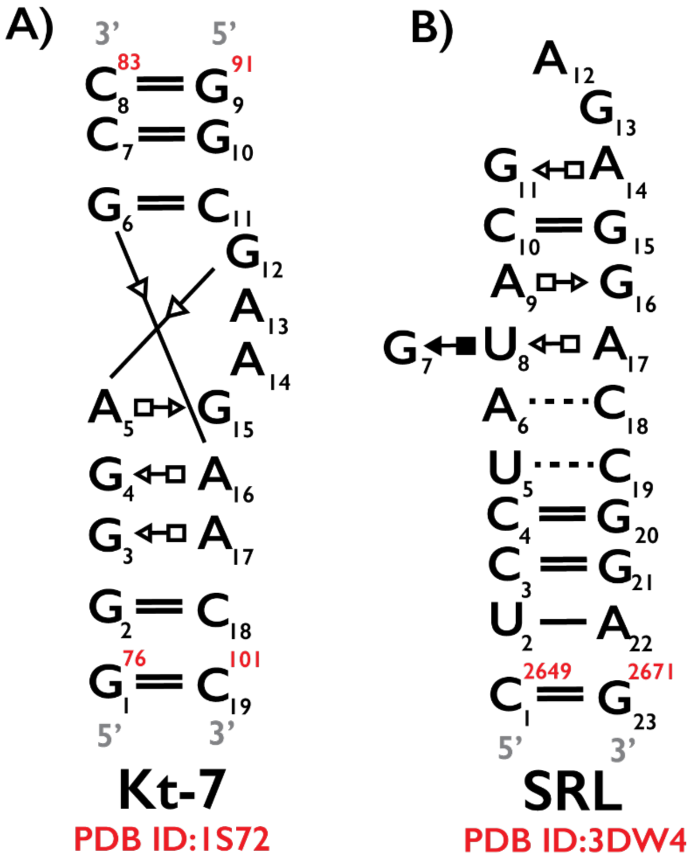

Figure S7: Secondary structure annotation following the Leontis−Westhof nomenclature[14] for RNA kink-turn 7 (Kt-7, panel A) and Sarcin-Ricin loop (SRL, panel B) motifs. As starting coordinates for both those motifs were excised from larger experimental structures (see Methods in main text), we are showing both original (red) and relabeled (black) residue indexes. Starting coordinates are also attached (zipped PDB files).

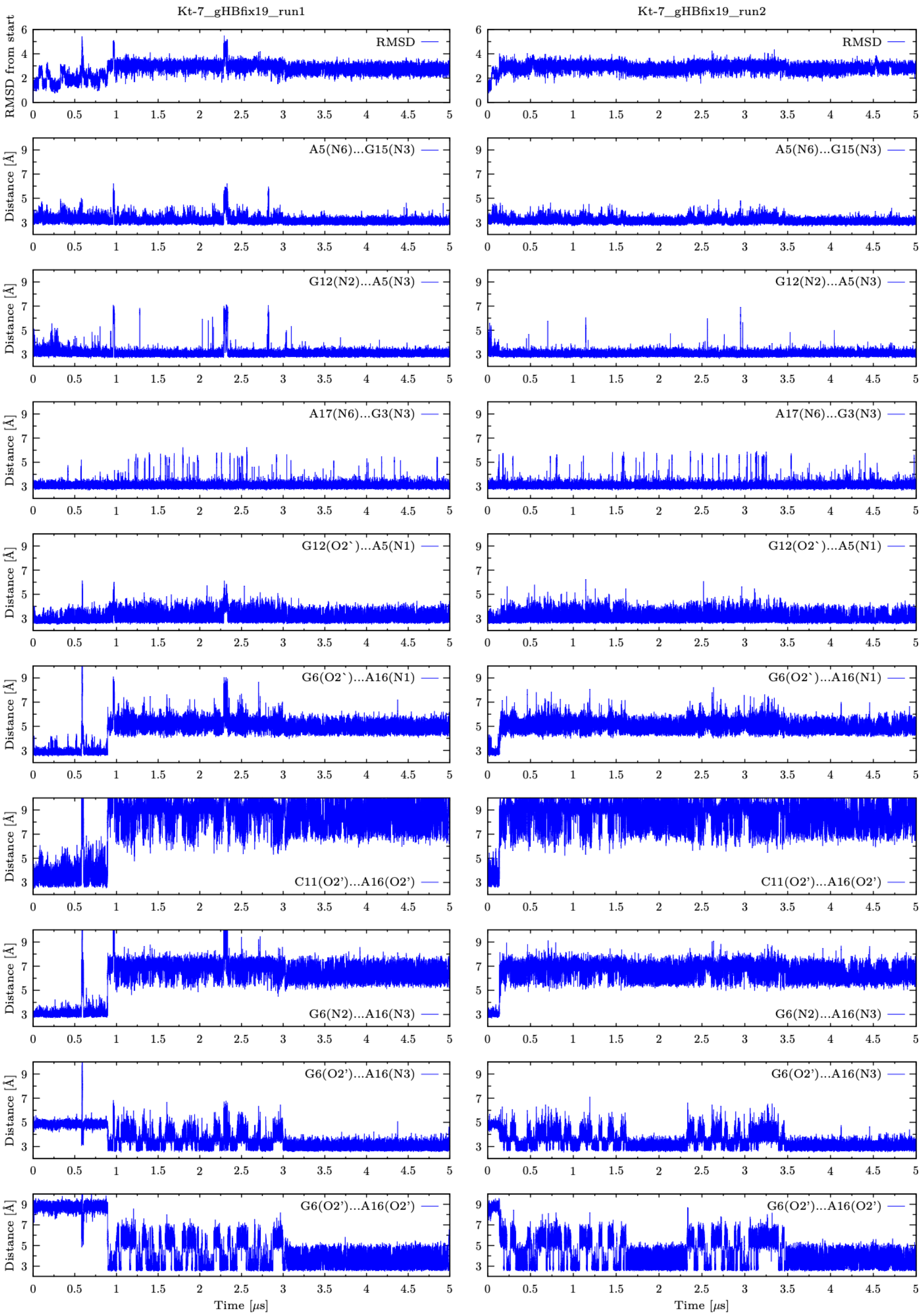

Figure S8: Structural analysis of two independent control standard MD simulations of the Kt-7 motif using OL3$_{CP}$ *ff* with gHBfix19 potential (see Methods in main text and Table S2). Plots on the top are showing fluctuations of RMSD calculated over heavy atoms of all nucleotides during 5 µs-long unbiased MD simulations. Remaining plots are displaying fluctuations of key distances.

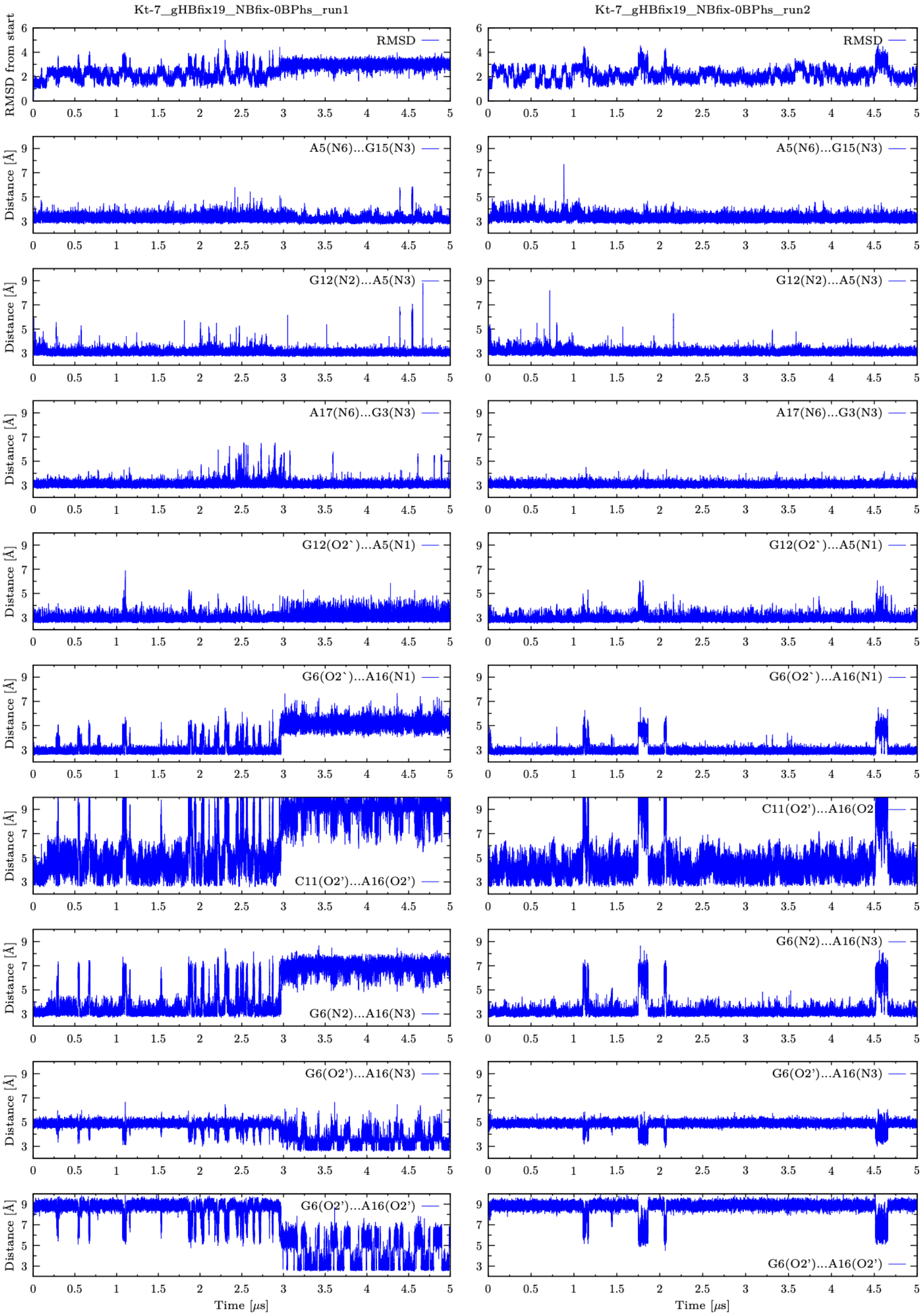

Figure S9: Structural analysis of two independent standard MD simulations of Kt-7 motif using $OL3_{CP}$ *ff* with gHBfix19 potential and $NBfix_{0BPh}$ modification (see legend of Figure S8 for more details).

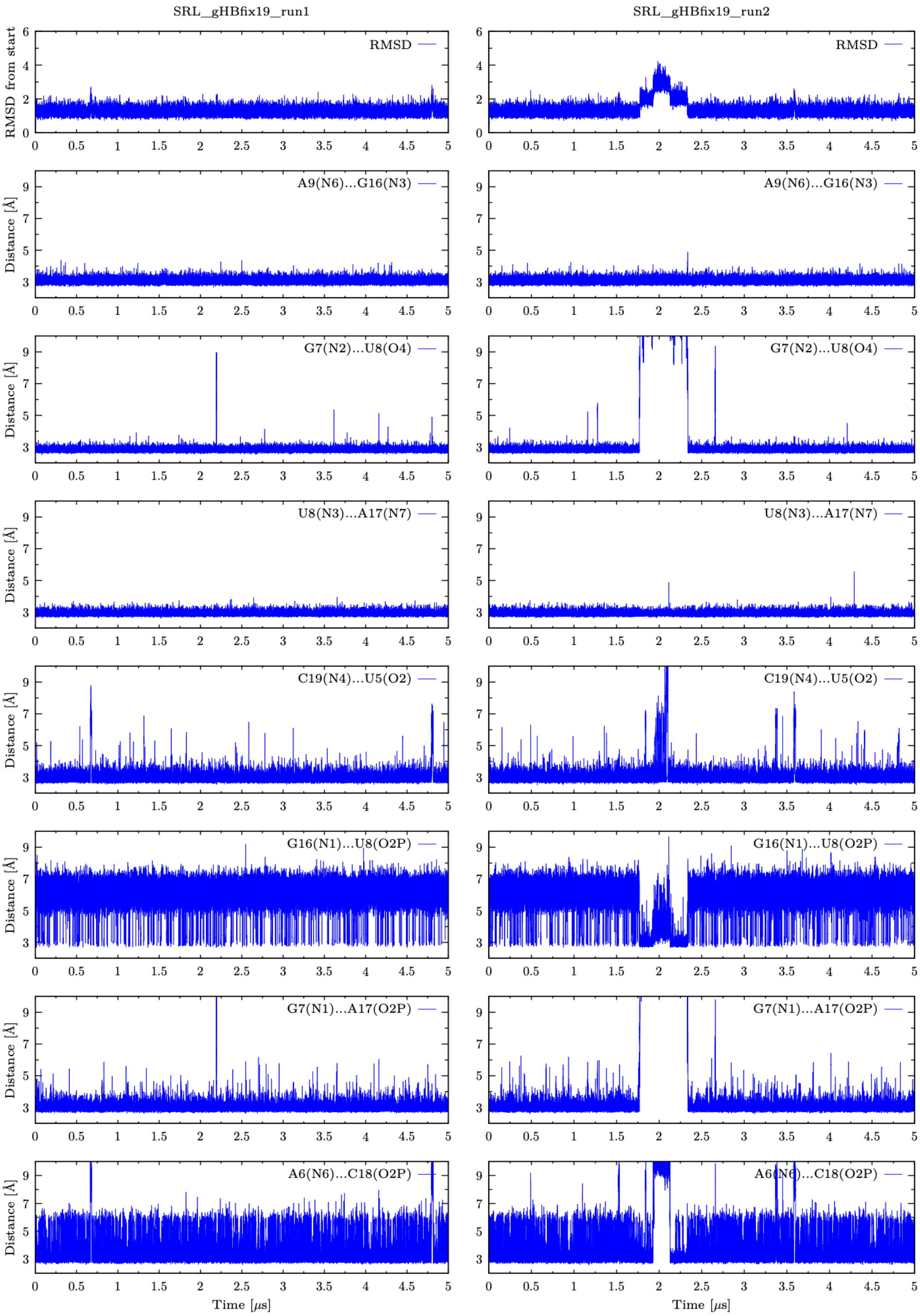

Figure S10: Structural analysis of two independent standard MD simulations of SRL motif using OL3$_{CP}$ *ff* with gHBfix19 potential (see Methods in main text and Table S2). Plots on the top are showing fluctuations of RMSD calculated over heavy atoms of all nucleotides during 5 μs-long unbiased MD simulations. Remaining plots are displaying fluctuations of key distances.

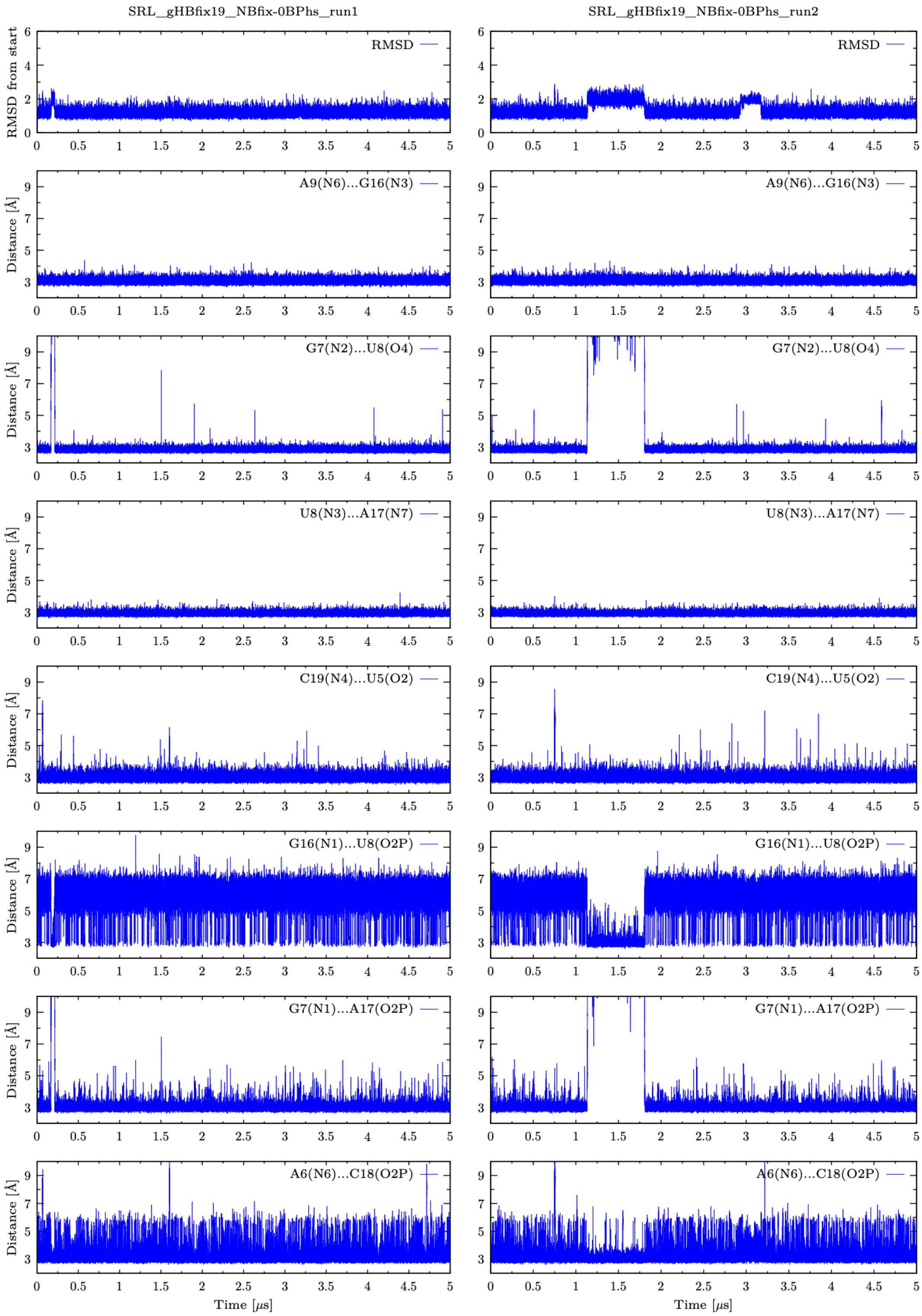

Figure S11: Structural analysis of two independent standard MD simulations of SRL motif using OL3$_{CP}$ *ff* with gHBfix19 potential and NBfix$_{0BPh}$ modification (see legend of Figure S10 for more details).

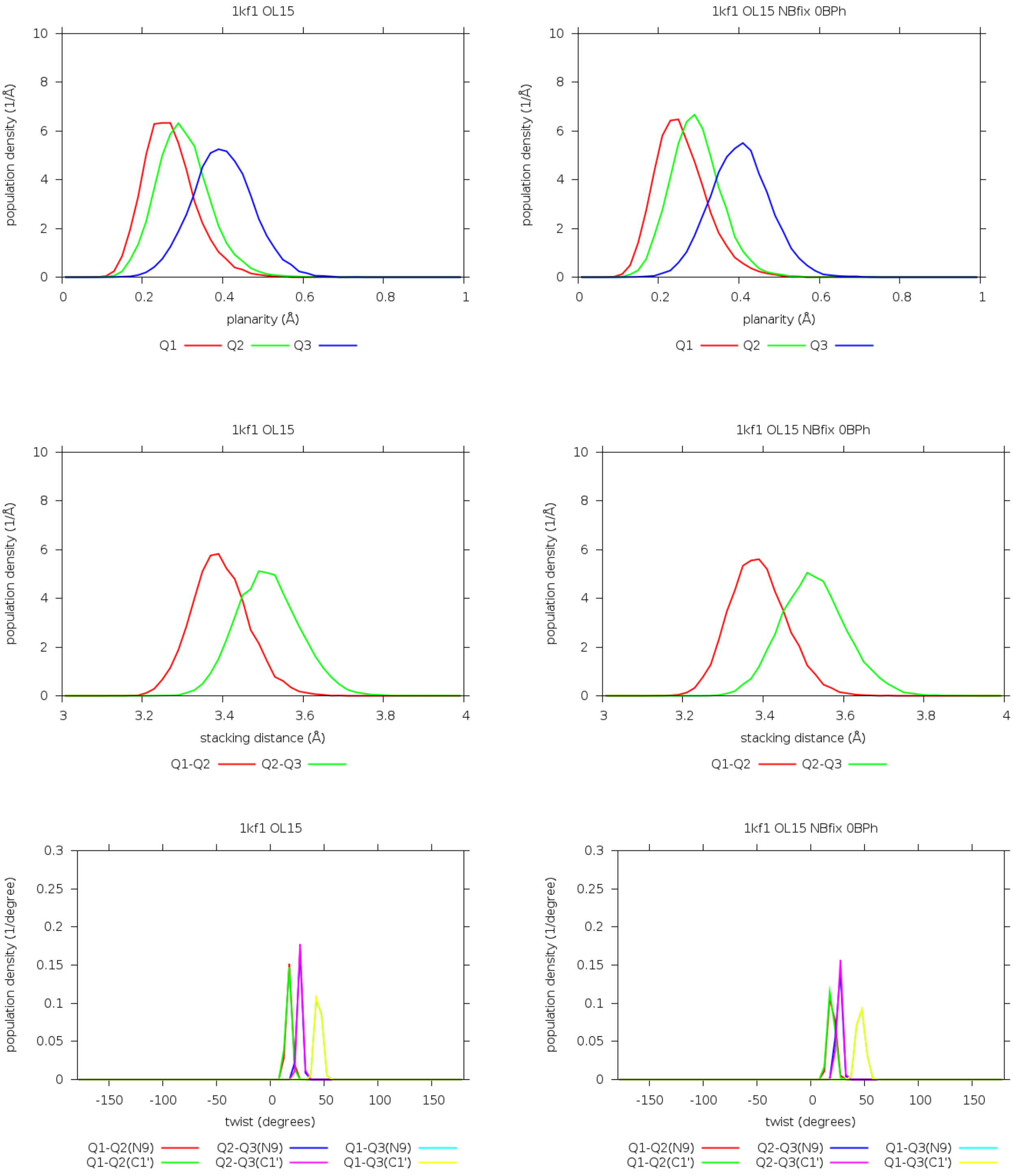

Figure S12: GQ structural parameters observed in the simulations of parallel-stranded 1KF1 GQ. The graphs show essentially identical (within the limits of sampling) distributions of quartet planarities (top row; Q1 – 5'-quartet, Q2 – middle quartet, Q3 – 3'quartet), stacking distances (middle row) and helical twists (bottom row; measured either by the position of C1' atoms or N9 atoms) between adjacent quartets in the standard OL15 *ff* (control; left column) and the version with added NBfix$_{0BPh}$ term (right column). Planarity of a quartet was defined as the root mean square deviation of the distances of non-hydrogen atoms to the best-fit quartet plane. Stacking distance between two quartets was calculated as a sum of distances of the two quartet centers to the quartets' best-fit planes average.[15]

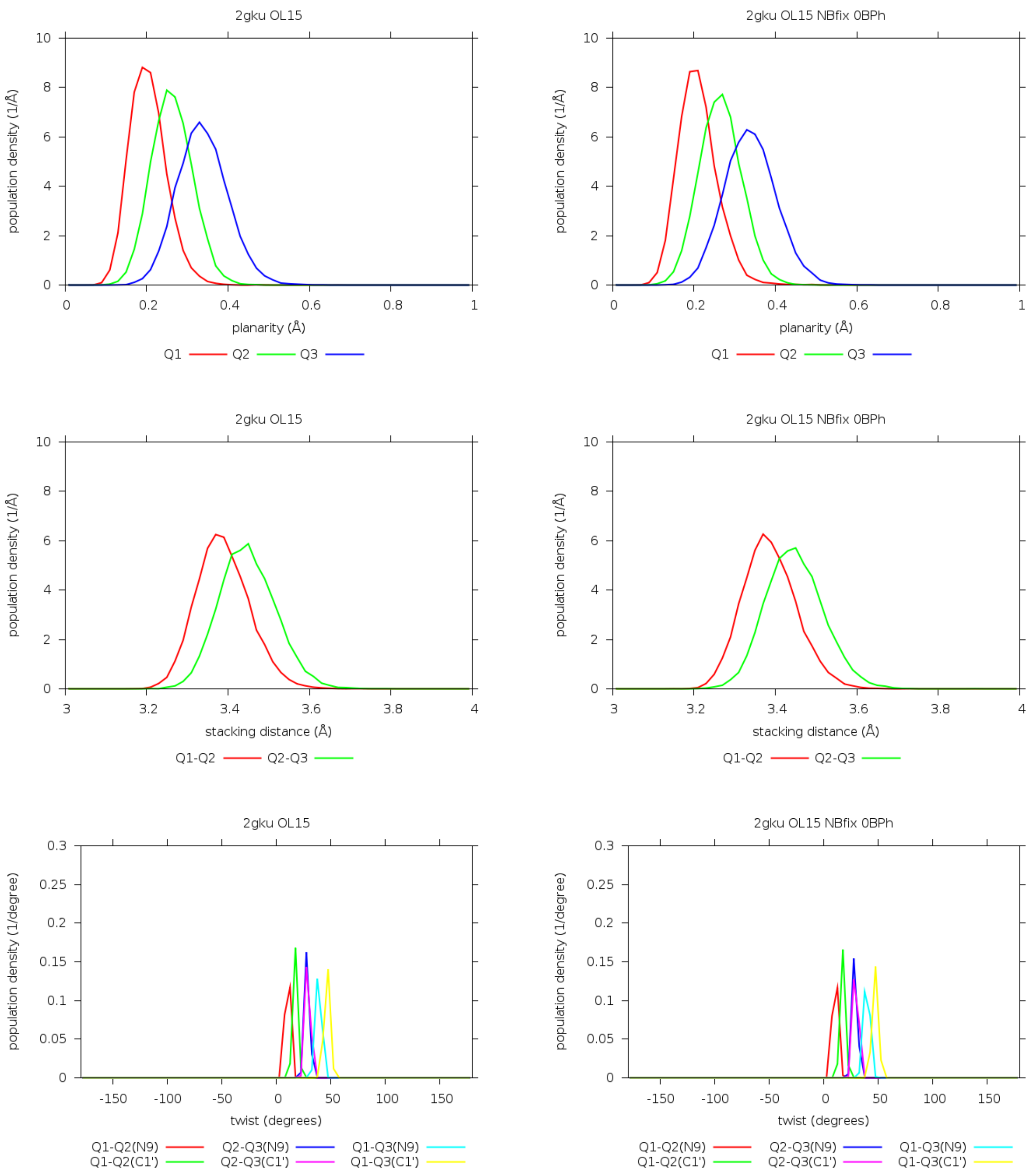

Figure S13: GQ structural parameters observed in the simulations of (3+1) hybrid 2GKU GQ. The graphs show identical (within the limits of sampling) outcome between the standard OL15 *ff* (control; left column) and the version with $NBfix_{0BPh}$ term (right column). See legend of Figure S12 for more details.

REFERENCES

(1) Kuhrova, P.; Mlynsky, V.; Zgarbova, M.; Krepl, M.; Bussi, G.; Best, R. B.; Otyepka, M.; Sponer, J.; Banas, P. Improving the Performance of the Amber RNA Force Field by Tuning the Hydrogen-Bonding Interactions. *J Chem Theory Comput* **2019**, *15* (5), 3288-3305. DOI: 10.1021/acs.jctc.8b00955.
(2) Klosterman, P. S.; Shah, S. A.; Steitz, T. A. Crystal structures of two plasmid copy control related RNA duplexes: An 18 base pair duplex at 1.20 A resolution and a 19 base pair duplex at 1.55 A resolution. *Biochemistry* **1999**, *38* (45), 14784-14792.
(3) Dockbregeon, A. C.; Chevrier, B.; Podjarny, A.; Johnson, J.; Debear, J. S.; Gough, G. R.; Gilham, P. T.; Moras, D. Crystallographic Structure of an Rna Helix - [U(Ua)6a]2. *J Mol Biol* **1989**, *209* (3), 459-474.
(4) Zgarbova, M.; Otyepka, M.; Sponer, J.; Lankas, F.; Jurecka, P. Base Pair Fraying in Molecular Dynamics Simulations of DNA and RNA. *J Chem Theory Comput* **2014**, *10* (8), 3177-3189. DOI: 10.1021/ct500120v.
(5) Banas, P.; Hollas, D.; Zgarbova, M.; Jurecka, P.; Orozco, M.; Cheatham, T. E.; Sponer, J.; Otyepka, M. Performance of Molecular Mechanics Force Fields for RNA Simulations: Stability of UUCG and GNRA Hairpins. *J Chem Theory Comput* **2010**, *6* (12), 3836-3849, Article. DOI: 10.1021/ct100481h.
(6) Drew, H. R.; Wing, R. M.; Takano, T.; Broka, C.; Tanaka, S.; Itakura, K.; Dickerson, R. E. Structure of a B-DNA dodecamer: conformation and dynamics. *Proc Natl Acad Sci U S A* **1981**, *78* (4), 2179-2183. DOI: 10.1073/pnas.78.4.2179.
(7) Reblova, K.; Sponer, J. E.; Spackova, N.; Besseova, I.; Sponer, J. A-minor tertiary interactions in RNA kink-turns. Molecular dynamics and quantum chemical analysis. *J Phys Chem B* **2011**, *115* (47), 13897-13910. DOI: 10.1021/jp2065584.
(8) Matouskova, E.; Drsata, T.; Pfeifferova, L.; Sponer, J.; Reblova, K.; Lankas, F. RNA kink-turns are highly anisotropic with respect to lateral displacement of the flanking stems. *Biophys J* **2022**, *121* (5), 705-714. DOI: 10.1016/j.bpj.2022.01.025.
(9) Parkinson, G. N.; Lee, M. P.; Neidle, S. Crystal structure of parallel quadruplexes from human telomeric DNA. *Nature* **2002**, *417* (6891), 876-880. DOI: 10.1038/nature755.
(10) Luu, K. N.; Phan, A. T.; Kuryavyi, V.; Lacroix, L.; Patel, D. J. Structure of the human telomere in K+ solution: an intramolecular (3 + 1) G-quadruplex scaffold. *J Am Chem Soc* **2006**, *128* (30), 9963-9970. DOI: 10.1021/ja062791w.
(11) Mlynsky, V.; Kuhrova, P.; Kuhr, T.; Otyepka, M.; Bussi, G.; Banas, P.; Sponer, J. Fine-Tuning of the AMBER RNA Force Field with a New Term Adjusting Interactions of Terminal Nucleotides. *J Chem Theory Comput* **2020**, *16* (6), 3936-3946. DOI: 10.1021/acs.jctc.0c00228.
(12) Mlynsky, V.; Janecek, M.; Kuhrova, P.; Frohlking, T.; Otyepka, M.; Bussi, G.; Banas, P.; Sponer, J. Toward Convergence in Folding Simulations of RNA Tetraloops: Comparison of Enhanced Sampling Techniques and Effects of Force Field Modifications. *J Chem Theory Comput* **2022**, *18* (4), 2642-2656. DOI: 10.1021/acs.jctc.1c01222.
(13) Zhao, J.; Kennedy, S. D.; Turner, D. H. Nuclear Magnetic Resonance Spectra and AMBER OL3 and ROC-RNA Simulations of UCUCGU Reveal Force Field Strengths and Weaknesses for Single-Stranded RNA. *J Chem Theory Comput* **2022**, *18* (2), 1241-1254. DOI: 10.1021/acs.jctc.1c00643.
(14) Leontis, N. B.; Westhof, E. Geometric Nomenclature and Classification of RNA Base Pairs. *RNA* **2001**, *7* (4), 499-512. DOI: 10.1017/s1355838201002515.
(15) Zhang, Z.; Mlynsky, V.; Krepl, M.; Sponer, J.; Stadlbauer, P. Tetrameric Parallel Guanine-Quadruplex Folding Pathways Unveiled by Steered Molecular Dynamics Simulations. *manuscript in preparation*.
